# Whole-exome sequencing in deceased fetuses with ultrasound anomalies: a retrospective analysis

**DOI:** 10.1186/s12920-022-01427-1

**Published:** 2023-02-16

**Authors:** Wei Huang, Xiaofan Zhu, Gege Sun, Zhi Gao, Xiangdong Kong

**Affiliations:** Department of Obstetrics and Gynecology, Genetics and Prenatal Diagnosis Center, The First Affiliated Hospital of Zhengzhou University, Zhengzhou, 450052 China

**Keywords:** Whole-exome sequencing, Deceased fetuses, Products of conception, Genetic diagnosis

## Abstract

**Background:**

Whole-exome sequencing (WES) is an effective method in the prenatal setting for identification of the underlying genetic etiology of fetal ultrasound abnormalities. To investigate the diagnostic value of WES in fetuses with ultrasound abnormalities that resulted in fetal demise or pregnancy termination.

**Methods:**

61 deceased fetuses with ultrasound abnormalities and normal copy number variation Sequencing were retrospectively collected. Proband-only or trio-WES were performed on the products of conception.

**Result:**

Collectively, 28 cases were positive with 39 variants (10 pathogenic, 22 likely pathogenic and 7 variants of uncertain significance) of 18 genes, and the overall diagnostic rate was 45.9% (28/61), of which 39.2% (11/28) were de novo variants. In addition, 21 variants in 11 genes among the positive cases had not been previously reported. The diagnostic yield for definitive findings for trio analysis was 55.9% (19/34) compared to 33.3% (9/27) for singletons. The most common ultrasound abnormalities were skeletal system abnormalities 39.2% (11/28), followed by multiple system abnormalities (17.9%, 5/28) and genitourinary abnormalities (17.9%, 5/28).

**Conclusion:**

Our results support the use of WES to identify genetic etiologies of ultrasound abnormalities and improve understanding of pathogenic variants. The identification of disease-related variants provided information for subsequent genetic counseling of recurrence risk and management of subsequent pregnancies.

## Background

Congenital structural abnormalities are identified in approximately 3% of fetuses, accounting for 25% of perinatal deaths [1, 2]. Fetal structural abnormalities can vary from isolated minor anomalies to severe multi-system abnormalities, which can be effectively identified by prenatal ultrasound [3]. The identification of fetal ultrasound anomalies prompts additional prenatal evaluations. Most of the structural abnormalities indicated by prenatal ultrasound occur in fetuses with no family history of congenital malformation, making accurate prenatal genetic counselling difficult. Therefore, it is necessary to clarify the genetic etiology of fetal structural abnormalities. Chromosomal abnormalities and monogenic disorders have been considered significant causes of congenital defects, although the etiology of many congenital malformations is unknown. G-banded karyotyping identified numerical and structural chromosomal abnormalities in 50% of fetuses with ultrasound abnormalities, low-coverage genome sequencing (e.g. CNV-seq) increases the detection rate in this group of fetuses by up to 5% [4, 5]. However, the identifiable genetic cause is still undetected in over 60% of cases. Whole-exome sequencing (WES) can identify the genetic etiology by identifying the single gene pathogenic variation of fetal structural abnormalities. According to previous studies, WES can be used to discover the genetic causes of fetal structural abnormalities, identify pathogenic variations, and establish genotype-phenotypic associations [6]. Therefore, the method of WES was used in this study to analyze the 61 causes of fetal specimens terminated due to structural abnormalities indicated by ultrasound.

## Methods

### Subjects

Fetal WES cases were retrospectively collected from pregnancies with ultrasound anomalies terminated or resulting in fetal demise between October 2020 and May 2022. All cases had previously undergone CNV-seq, and no clinically significant variants were detected. Fetal phenotype information was obtained from prenatal ultrasound and fetal magnetic resonance imaging reports. All the couples were not consanguineous. Early pregnancy was excluded. (gestational age < 12 weeks). Pregnant women with known teratogen, uterine malformation, hypertension, diabetes, and other basic diseases were also excluded. Ethics approval to undertake the research was granted by the First Affiliated Hospital of Zhengzhou University Committee.

### Sequencing analysis and variant annotation of WES

Fetal genomic DNA was obtained from products of conception (POC), including chorionic villi and fetal skin tissues. Parental DNA was extracted from peripheral blood samples. The genomic DNA of deceased fetuses and their parents were extracted using the QIAamp DNA extraction kit (Qiagen, Germany), then quantified using Qubit 4.0 (Thermo Fisher Scientific Inc. USA). DNA libraries were established by using Ada & Index Kit (UDI for LIM) and Enzyme Plus Library Prep Kit (iGeneTech Co., Ltd, Beijing, China), and exome coding and splicing regions were captured using AIExome Human Exome Panel V2 Plus with TargetSeq One Hyb & Wash Kit (iGeneTech Co., Ltd, Beijing, China), according to the manufacturer’s instructions. Subsequently, the captured libraries were sequenced on the NovaSeq6000 platform (Illumina, San Diego, CA, USA) with an average sequencing depth > 100X and 20X sequencing coverage > 98%. Sentieon(release 201,808.05) was used to align paired-end reads with the human reference genome GRCh37/hg19 under the Genome Analysis Toolkit(GATK) best practice guidelines, and the duplicate reads were removed using Picard version 2.9.0. Candidate variants were assessed with the latest reports in online databases, such as ClinVar, Human exons database (ExAC), Human Gene Mutation Database (HGMD) and Online Mendelian Inheritance in Man (OMIM) databases.

### Variant interpretation and classification

Variants were classified into pathogenic variants (P), likely pathogenic variants (LP), variants of uncertain significance (VUS), likely benign variants, and benign variants according to the guidelines of the American College of Medical Genetics and Genomics(ACMG) [7]. The P and LP variants were further analyzed with prenatal imaging to determine whether they fully and partially explain the observed phenotype. Polymerase chain reaction (PCR) combined with Sanger sequencing was used to validate the candidate P/LP variants in specimens of deceased fetuses and peripheral blood of parents. Clinical reports were provided to the families, including P and LP variants associated with the ultrasound phenotype and VUS variants highly consistent with the prenatal ultrasound phenotype.

## Results

### Demographic characteristics

**Fig. 1:**
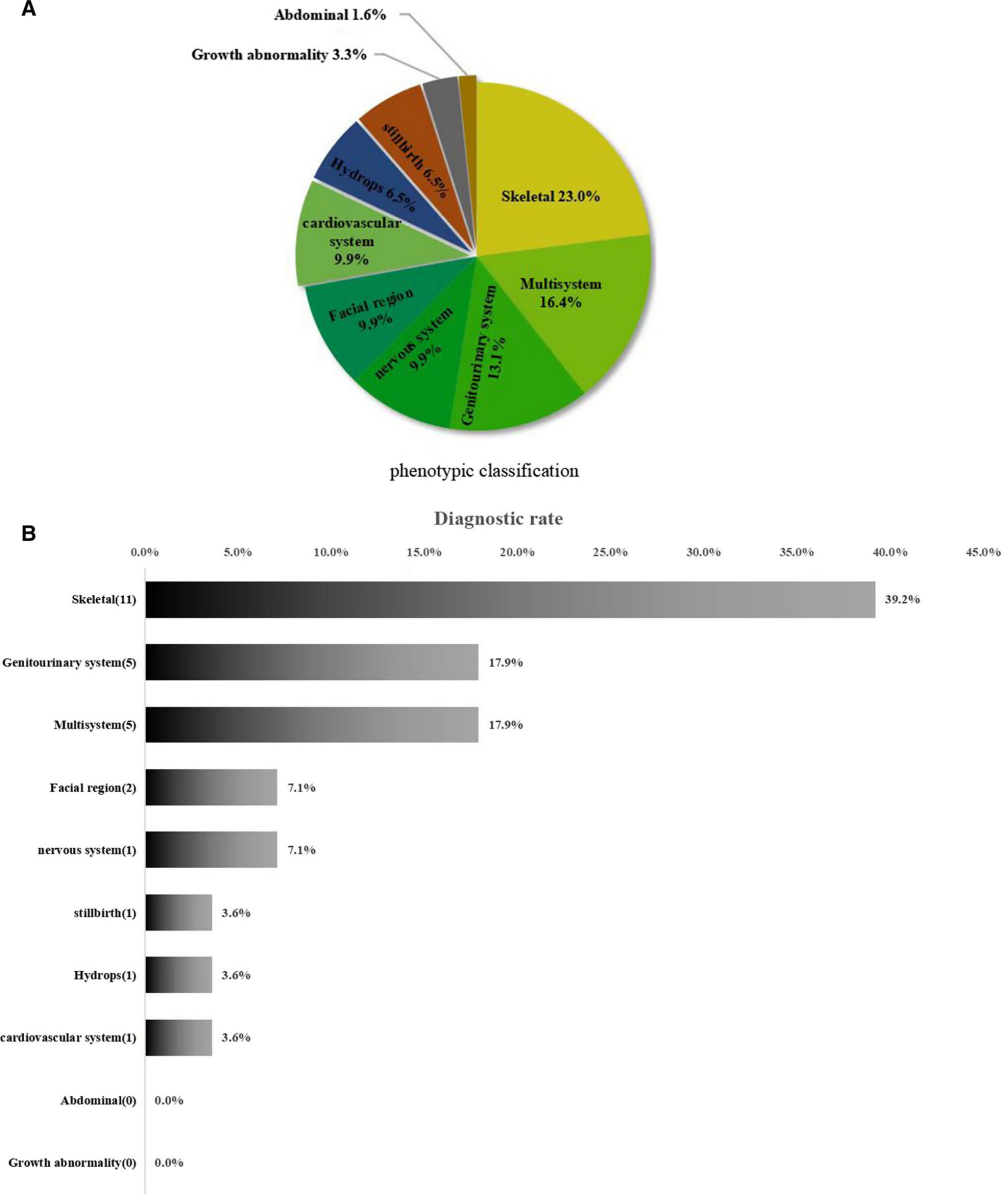
Phenotypic spectrum of fetuses with ultrasound anomalies undergoing ES. **A** Scaled representation of relative frequency of each phenotype class within this series. **B** Diagnostic rate for each phenotype class in positive case

We analyzed WES results from 61 deceased fetuses, with 44.3% (27/61) submitted as singleton-only and 55.7% (34/61) as proband-parent trios. Among single-only, the most common abnormality was the skeletal system, while among proband-parent trios, the most common abnormality was the genitourinary system, followed by the skeletal system. All cases terminated the pregnancies due to severe ultrasound abnormalities. The maternal age was 22 to 38 years old (median 30 years); the mean gestational age was 21 weeks, ranging from 12 to 33 weeks and 1 day. Cases are most common in the second trimester. We summarized detailed gestational age information of the diagnostic cases in Table 1. A total of 15 women (25%) had previous pregnancy histories of fetus with similar congenital malformations. There were 47.5% (N = 29) males and 52.5% (N = 32) females based on fetal sex determined by WES data. The 61 cases were categorized into 10 classes according to the anatomical system affected, including skeletal system (14, 23.0%), multisystem (10, 16.4%), genitourinary (8, 13.1%), nervous system (6, 9.9%), facial region (6, 9.9%), cardiovascular (6, 9.9%), hydrops (4, 6.5%), stillbirth (4, 6.5%), growth abnormality (2, 3.3%), and abdominal (1, 1.6%) (Fig. 1A).

**Table 1:** Distribution of gestational age observed across 28 molecular diagnoses in 61 cases

Gestational age	Number of diagnoses	Percent of diagnoses (%)
< 14 W	2	7.2
≥ 14 W, < 28 W	23	82.1
≥ 28 W	3	10.7

### Potential diagnostic variants and detection rate of WES in deceased fetuses

Among the 61 cases included, 28 cases yielded a diagnosis by WES with a detection rate of 45.9% (28/61), involving 39 variants in 18 genes (10 P, 22, and 7 VUS). Detailed phenotypic and variant information of the diagnostic cases were summarized in Table 2. Skeletal system abnormalities yielded the highest detected rate of 39.2% (11/28), followed by 17.9% (5/28) in multi-system, 17.9% (5/28) in genitourinary abnormalities, 7.1% (2/28) in facial abnormalities, 7.1% (2/28) in the nervous system, and 3.6% (1/28) in fetal hydrops, cardiovascular system abnormalities, and stillbirth, respectively (Fig. 1B). The diagnostic rate was 48.3% (14/29) for male fetuses and 43.8% (14/32) for female fetuses. The difference was not statistically significant (p = 0.723). The diagnostic rate of single system abnormalities was 45.1% (23/51), and that of two or more systems abnormalities was 50% (5/10). The diagnostic rate of two or more systems of fetal abnormalities was slightly higher than that of single-system, but the difference was not statistically significant (p = 0.776).

**Table 2:** Overview of cases with positive findings explanatory of the fetal phenotypes

Case No	GA (weeks)	Ultrasound finding(Hpo number)	Gene(OMIM)	Variant	Variant classification (ACMG criteria)	Inheritance mode	Disease	Origin
1	24	Polycystic kidney dysplasia (0,000,113)	*PKHD1*(263,200)	NM_138694:c.8301delC(p.Asn2768fs) het Exons 58–60 repeat het	LP(PVS1 + PM2) LP	AR	Polycystic Kidney Disease 4 With Or Without Polycystic Liver Disease	Maternal/paternal
2	24	Polycystic kidney dysplasia (0,000,113) Oligohydramnios (0,001,562)	*JAG1*(118,450)	NM_000214:c.2078_2079delGT(p.Cys693fs) het	P(PVS1 + PM2 + PP5)	AD	Alagille Syndrome 1	Maternal
3	14^+3^	Abnormality of long bone morphology (0,011,314) Medial deviation of the foot (0,008,082) Hydrops fetalis (0,001,789)	*COL1A1*(114,000)	NM_000088:c.2399G > A(p.Gly800Glu) het	LP(PM1 + PM2 + PM6 + PP2 + PP3)	AD	Caffey disease	De novo
4	18^+6^	Abnormal thorax morphology (0,000,765) Aplasia/hypoplasia of the extremities (0,009,815) Thickened nuchal skin fold (0,000,474)	*FGFR3* (100,800)	NM_000142:c.742C > T(p.Arg248Cys) het	P(PS4 + PM1 + PM2 + PP3)	AD	Achondroplasia	De novo
5	19	Short lower limbs (0,006,385)	*FGFR3* (100,800)	NM_000142:c.742C > T(p.Arg248Cys) het	P(PM1 + PM2 + PP3 + PS4)	AD	Achondroplasia	De novo
6	24^+1^	Aplasia/hypoplasia of the extremities (0,009,815) Abnormal thorax morphology (0,000,765)	*FGFR3* (100,800)	NM_000142:c.742C > T(p.Arg248Cys) het	P(PM1 + PM2 + PP3 + PS4)	AD	Achondroplasia	De novo
7	33^+1^	Abnormal thorax morphology (0,000,765) Aplasia/hypoplasia of the extremities (0,009,815)	*DYNC2H1* (613,091)	NM_001080463:c.9182_9185delAGAG(p.Glu3061fs) het c.7495C > G(p.Leu2499Val) het	LP(PVS1 + PM2_S) VUS(PM1 + PM2_S + PM3)	AR	Asphyxiating Thoracic Dystrophy 3	Maternal/paternal
8	13	Micrognathia (0,000,347)	*GNAI3* (602,483)	NM_006496:c.119G > T(c.119G > T) het	LP(PM1 + PM2 + PM6 + PP3)	AD	Auriculocondylar Syndrome 1	De novo
9	24	Hydrocephalus (0,000,238)	*LICAM* (307,000)	NM_000425:c.1258delT(p.Tyr420fs)	LP(PVS1 + PM2_S)	XR	Hydrocephalus Due To Congenital Stenosis Of Aqueduct Of Sylvius	*Maternal*
10	29^+4^	Polycystic kidney dysplasia (0,000,113) Polyhydramnios (0,001,561)	*PKHD1* (263,200)	NM_138694:c.979A > G(p.Asn327Asp) het c.11282C > A(p.Gln3761Lys) het	VUS(PM1 + PM2) VUS(PM2 + PP3)	AR	Polycystic Kidney Disease 4 With Or Without Polycystic Liver Disease	Maternal/paternal
11	16	Short long bone (0,003,026)	*COL1A1* (166,200)	NM_000088:c.1273G > A(p.Gly425Ser) het	LP(PM1 + PM2_S + PM6 + PP2 + PP3)	AD	Osteogenesis Imperfecta, Type I	De novo
12	23	Hyperechogenic kidneys (0,004,719)	*ACE* (267,430)	NM_000789:c.1028G > A(p.Trp343*) hom	P(PVS1 + PM2_S + PM3)	AR	Renal Tubular Dysgenesis	Maternal/paternal
13	17^+3^	Aplasia/hypoplasia of the extremities (0,009,815) Abnormal thorax morphology (0,000,765) Polydactyly (0,010,442)	*DYNC2H1* (613,091)	NM_001080463:c.557G > T(p.Gly186Val) het c.7643 T > C(p.Phe2548Ser) het	VUS(PM2 + PM3) LP(PS1 + PM2 + PM3)	AR	Asphyxiating Thoracic Dystrophy 3	Maternal/paternal
14	14^+^	Short long bone (0,003,026) Abnormal thorax morphology (0,000,765) Hypoplasia of the frontal bone (0,005,466) Large fontanelles (0,000,239)	*DYNC2H1* (613,091)	NM_001080463:c.8190G > T(p.Leu2730Phe) het c.8621delC(p.Leu2876fs*15) het	LP(PVS1 + PM2) LP(PVS1 + PM2)	AR	Asphyxiating Thoracic Dystrophy 3	Maternal/paternal
15	25^+^	Hydrocephalus (0,000,238) Dilation of lateral ventricles (0,006,956) Absent septum pellucidum (0,001,331)	*L1CAM* (307,000)	NM_000425:c.1989C > G(p.Tyr663*) hemi	LP(PVS1 + PM2)	XR	Hydrocephalus Due To Congenital Stenosis Of Aqueduct Of Sylvius	Maternal
16	21^+2^	Abnormal thorax morphology (0,000,765) Aplasia/hypoplasia of the extremities (00,098,150)	*DYNC2H1* (613,091)	NM_001080463:c.2070A > T(p.Arg690Ser) het c.8946 + 1G > A het	VUS(PM2_S + PM3) LP(PVS1 + PM2)	AR	Asphyxiating Thoracic Dystrophy 3	Maternal/paternal
17	23	Medial deviation of the foot (0,008,082) Abnormal hand morphology (0,005,922)	*NEB* (619,334)	NM_001271208:c.25159C > T(p.Gln8387*) het c.7494dupT(p.Asp2499fs) het	LP(PVS1 + PM2_S + PP4) LP(PVS1 + PM2_S + PP4)	AR	Arthrogryposis Multiplex Congenita 6	Maternal/paternal
18	12^+4^	Aplasia/Hypoplasia of the radius (0,006,501)	*SALL4* (607,323)	NM_001318031:c.844delC(p.Gln282fs) het	P(PVS1 + PM2 + PP1)	AD	Duane-radial ray syndrome	Paternal
19	19^+4^	Hydronephrosis (0,000,126) Abnormality of bladder morphology (0,025,487) Rocker bottom foot (0,001,838)	*CAD* (616,457)	NM_001306079:c.2665_2666insCG(p.Lys889fs) het c.6055G > A(p.Asp2019Asn) het	LP(PVS1 + PM2) VUS(PM2 + PP2 + PP3)	AR	Epileptic Encephalopathy, Early Infantile, 50	Maternal/paternal
20	23	Skeletal dysplasia (0,002,652) Abnormality of cardiovascular system morphology(0,030,680) Renal hypoplasia (0,000,089)	*NOTCH2* (610,205)	NM_024408:c.6973C > T(p.Gln2325*)	P(PVS1 + PS2 + PM2_S)	AD	Alagille Syndrome 2	De novo
21	24	Talipes equinovarus (0,001,762)	*ECEL1* (615,065)	NM_004826:c.1700C > G(p.Pro567Arg) het c.922C > T(p.Gln308*)	VUS(PM3 + PM2_S + PP3) LP(PVS1 + PM2_S)	AR	Arthrogryposis, Distal, Type 5d	Maternal/paternal
22	26	Stillbirth (0,003,826)	*PLAA* (617,527)	NM_001031689:c.1658-2A > G het c.1043delC(p.Thr348fs) het	LP(PVS1 + PM2_S + PP3) LP(PVS1 + PM2_S)	AR	Neurodevelopmental Disorder With Progressive Microcephaly, Spasticity, And Brain Anomalies	Maternal/paternal
23	18	Abnormal lower limb bone morphology (0,040,069)	*COL1A1* (114,000)	NM_000088:c.3150_3158dupTCCTGGTGC(p.Ala1053_Pro1054insProGlyAla) het	LP(PM2_S + PM4 + PM6)	AD	Caffey disease	De novo
24	25	Polycystic kidney dysplasia (0,000,113)	*PKHD1* (263,200)	NM_138694:c.3305_3306delAT(p.Tyr1102fs) het c.5935G > A(p.Gly1979Arg) het	P(PVS1 + PM2_S + PP4) LP(PM3 + PM2_S + PM1 + PP3 + PP4)	AR	Polycystic Kidney Disease 4 With Or Without Polycystic Liver Disease	Maternal/paternal
25	24	Cleft upper lip (0,000,204) Cleft palate (0,000,175)	*TFAP2A* (113,620)	NM_001372066:c.890-1G > A	P(PVS1 + PM2)	AD	Branchiooculofacial Syndrome	De novo
26	13^+3^	Cleft upper lip (0,000,204) Cleft palate (0,000,175)	*KAT6B* (606,170)	NM_001372066:c.4017delA(p.Gly1340fs) het	LP(PVS1 + PM2_S + PM6)	AD	Genitopatellar syndrome	De novo
27	24	Abnormal heart morphology (0,001,627)	*ARID1A* (614,607)	NM_006015:c.2402dupG(p.Gln802fs) het	LP(PVS1 + PM2)	AD	Coffin-siris Syndrome 2	De novo
28	19	Pleural effusion (0,002,202) Fetal choroid plexus cysts (0,011,426)	*PIEZO1* (616,843)	NM_001142864:c.5262delG(p.Trp1754fs) het c.1684C > T(p.Gln562*) het	P(PVS1 + PM2_S + PM3) LP(PVS1 + PM2)	AR	Lymphedema, Hereditary, Iii	Maternal/paternal

*GA*, Gestational age; *HPO*, Human Phenotype Ontology, pathogenic; *LP*, Likely pathogenic; *VUS*, Variant of unknown significance; *PM2_S*, PM2_supporting; *AR*, Autosomal recessive; *AD*, Autosomal dominant; *XLR*, X-linked recessive; *het*, heterozygous variant; *hemi*, hemizygous variant; *hom*, homozygous variant

### Inheritance mode in the diagnosed cases

Among the 28 cases with diagnostic results, 46.45% (13/28) were associated with autosomal dominant (AD) diseases, 46.45% (13/28) with autosomal recessive (AR) conditions, and 7.1% (2/28) with X-linked recessive diseases. A total of 11 variants had arisen de novo (3 cases with missense variant in *FGFR3*, 2 cases with missense variant in *COL1A1*, 4 cases with frameshift variant in *SALL4*, *KAT6B*, *ARIDA1* and *COL1A1*, 1 case with nonsense variant in *NOTCH*2, 1 case with missense variant in *GNAI3*), all of them were associated with AD conditions. In the remaining 15 cases with recessive pathogenic variants, 12 cases (80%) had biparentally inherited compound heterozygous variants, 1 case had a homozygous variant (missense variant in *ACE*) and 2 fetuses inherited the variant in chromosome X from the mother. In particular, in Case No.9, supratententate hydrocephalus with narrow transparent compartments was detected by ultrasound. A heterozygous variant of *L1CAM* was found in this female fetus by WES, which was inherited from the mother. The phenotype was consistent with X-linked cerebral edema (HSAS) caused by this gene. Although the condition was X-linked recessive inheritance, considering that similar fetal malformations were found in two previous pregnancies, the phenotypes in the current fetus may be caused by skewed X-inactivation. But further studies are needed to confirm this. In addition, 21 novel variants in 11 genes were found. The inheritance mode of 28 cases was listed in Table3.

**Table 3:** Models of inheritance observed across 28 molecular diagnoses in 61 cases

Mode of inheritance	Number of diagnoses	Percent of diagnoses	Number of novel variants
*Autosomal dominant*	13	46.45%	4
De novo	11		2
Inherited maternal	1		
Inherited,paternal	1		2
*Autosomal recessive*	13	46.45%	17
Homozygous	1		
Compound heterozygous	12		17
*X-linked*	2	7.1%	
Inherited maternal	2		

### Disease categories involved in the diagnosed cases

The most prevalent disease was skeletal disorders involving six genes (*FGFR3*, *DYNC2H1*, *COL1A1*, *NEB*, *SALL4*, *ECEL1*) in 11 cases. *FGFR3* associated with achondroplasia (100,800) were identified in 3 cases, 4 cases of Asphyxiating Thoracic Dystrophy 3 (613,091) were caused by *DYNC2H1*. In the remaining 4 cases, each was found with P/LP variants in *COL1A1* associated with Osteogenesis Imperfecta, Type I (166,200), *NEB* gene associated with Arthrogryposis Multiplex Congenital 6 (619,334), *SALL4* associated with Duane-radial Ray Syndrome (607,323), *ECEL1* associated with Arthrogryposis, Distal, Type 5d (615,065), respectively. The second most common category was multi-system involving 5 genes-*COL1A1*, *FGFR3*, *CAD*, *NOTCH2*, *KAT6B*-in 5 cases. For the genitourinary system, variants in 3 genes-*PKHD1*, *JAG1*, *ACE*- were identified in 5 cases, among which 3 cases were Polycystic Kidney Disease 4 With Or Without Polycystic Liver Disease (263,200) caused by *PKHD1.* In the remaining 2 cases, each was found with P/LP variants in *JAG1* gene associated with Alagille Syndrome 1 (118,450), *ACE* gene associated with Renal Tubular Dysgenesis (267,430). Variants in 2 genes-*GNAI3*, *TFAP2A-*associated with facial region were identified in two cases. *GNAI3* gene associated with Auriculocondylar Syndrome 1 (602,483), *TFAP2A* gene associated with Branchiooculofacial Syndrome (113,620). In addition, variants were identified in the other 4 phenotype categories involving 4 genes, including 2 cases of *L1CAM* associated with Hydrocephalus Due To Congenital Stenosis Of Aqueduct Of Sylvius (307,000), *PLAA* associated with Neurodevelopmental Disorder With Progressive Microcephaly, Spasticity, and Brain Anomalies (617,527), *PIEZO1* associated with Lymphedema, Hereditary, Iii (616,843), and *ARID1A* associated with Coffin-siris Syndrome 2 (614,607).

In case NO.2, we received a definitive diagnosis of Alagille syndrome type 1 caused by heterozygous variation of the *JAG1* gene, which was an autosomal dominant inheritance, and the variation came from the mother. Of the remaining 33 cases without a definitive diagnosis, there was one case with a VUS. Abnormal development of both kidneys was found in prenatal imaging of one case, and a heterozygous variant with the uncertain significance of *JAG1* was identified by singleton WES. Sanger sequencing indicated that the variant was inherited from the mother, who did not show any clinical phenotype.

## Discussion

WES can be used to explain the impact of monogenic disorders on pregnancy loss, elucidate the underlying genetic basis of structural developmental abnormality, establish a cause-effect relationship for fetal death, and enable a more accurate diagnosis of the disease. Currently, it is an effective prenatal method for identifying the underlying genetic etiology of fetal ultrasound abnormalities [8]. By using G-banded karyotyping, microarray analysis and WES, previous studies showed that the diagnostic rates of chromosomal abnormalities, pathogenic CNVs and monogenic variations in cases of fetal structural anomalies were 50%, 4%, and 22–36%, respectively [9]. These results suggest that with the addition of WES, current genetic testing can identify specific genetic etiology in about three quarters of deceased fetuses. In 61 fetal WES cases with ultrasound structural abnormalities, 39 variants of 18 genes were detected, and 28 cases received positive WES results, with a diagnosis rate of 45.9% (28/61).

Fu M et al. [10]. conducted WES of 19 aborted tissues and detected a total of 36 variation sequences, among which 12 were pathogenic variants, with a diagnosis rate as high as 33%. In another small study, Alamillo et al. [11]. reported seven fetal specimens from pregnancies with ultrasound anomalies in which three had “positive” results and one had a “likely positive” result, for a detection rate of 43% (3/7) to 57% (4/7). In these studies, the single-only was adopted, and the parental genetic factors were ignored, which limited the identification of recessive diseases. However, Elizabeth Quinlan-Jones et al. [12]. performed trio-WES in 27 deceased fetuses from induced labor or stillbirth due to ultrasound abnormalities, with a diagnostic rate of 37%, the phenotypic information was obtained from the prenatal imaging reports and the final autopsy report. In the study of Drury S et al. [13]. 14% of singleton cases had a positive result, which increased to 30% when trios were analyzed. Yates et al. [6]. performed WES in 84 deceased fetuses with structural anomalies with a diagnostic rate of 20%. In the Yates study, 52 performed parental/fetus trios with a diagnostic rate yield of 24%. In those probands with only fetal DNA tested, there was a lower diagnostic rate of 14%. This elevation of sensitivity is primarily due to the ability of performed parental/fetus trios to identify de novo variants and determine the phase for variants identified in recessive genes. In our study, we reported WES data in 61 cases with structural anomalies, singleton cases obtained a diagnostic yield of 33.3%, and this number increased to 55.9% when trios were analyzed. The improvement in diagnostic rates further proves that proband-parent trios were an effective strategy for identifying genetic factors of fetal ultrasound abnormalities. The disease categories in this study overlap considerably with those identified in previous studies of deceased fetuses, including multi-system diseases, urinary abnormalities, skeletal dysplasia, and central nervous system abnormalities. The data in our study confirmed the high diagnostic rate of trio-based WES in deceased fetuses with multi-system, genitourinary and skeletal system abnormalities. In our study, the diagnosis rate was higher than that of previous large-scale studies (22–36%) [9]. This may be because the cases in this cohort had serious structural malformations, showing characteristic ultrasonic phenotypes.

In this study, the diagnosis rate of multi-system abnormalities was higher than that of single-system abnormalities because the phenotype of fetal ultrasound was diverse, which could better establish the phenotypic and genotypic association. For example, case NO.4 was achondroplasia caused by a missense variant of the *FGFR3* gene. The ultrasound phenotype of this case not only included a narrow thoracic cavity but also thickened nuchal skin fold and dilated torcular herophili. This is consistent with the phenotypic diversity of the *FGFR3* gene. The most frequent ultrasound anomalies in the positive cases included skeletal systems, multi-system, nervous system and Genitourinary system anomalies. The most common diagnosis in this study was short-rib thoracic dysplasia type 3(SRTD3) with or without polydactyly caused by *DYNC2H1* in 5 cases. All these cases had similar prenatal ultrasound findings of hypoplasia of the extremities, with compound heterozygous variants identified in *DYNC2H1*. SRTD3 is a serious autosomal recessive fetal osteochondroplasia [14]. The *DYNC2H1* gene, located at 11q22.3, encodes a large cytoplasmic dynamin involved in the structure and function of cilia, which is involved in the retrograde transport of cilia and affects the formation of chondrocytes [15, 16]. *DYNC2H1* gene defects lead to the disruption of the Hedgehog signaling pathway, which affects the proliferation and differentiation of osteoblasts and chondrocytes, leading to chondroplasia [17, 18]. Bialleleic loss of function variants in this gene leads to severe fetal malformation, and a fatal condition whereby affected neonates die due to severe respiratory failure. Case NO.2 was found to have renal cystic dysplasia on prenatal ultrasonography and also demonstrated Oligohydramnios. WES analysis revealed a *JAG1* heterozygous splice variant consistent with a diagnosis of Alagille syndrome. *JAG1* gene can encode protein jagged-1 (JAG1) [19], a surface ligand in the highly conserved Notch signaling pathway, which can interact with the Notch receptor to regulate gene transcription [20]. The disease does not often lead to intrauterine death, it has well-defined postnatal phenotypes. More than 95 percent of ALGS patients develop heart defects. Butterfly vertebrae and specific facial features (triangular face, pointed chin) are also characteristic of Alagille syndrome [21, 22]. In addition to these features, most patients also have renal and vascular abnormalities. The current fetus showed renal abnormalities, and the variant was inherited from the mother, an imaging examination of the mother found that both kidneys were small accompanied by multiple cystic echoes. This suggests the effect of *JAG1* gene variant on renal development. However, abnormal growth of both kidneys was found in prenatal imaging of one negative case, and a heterozygous variant with unknown significance of *JAG1* was identified by WES. This variation was derived from the mother, but the mother did not have a similar clinical phenotype, which may be caused by the heterogeneity of the *JAG1* gene or the phenotypic diversity related to *JAG1* gene. In case No.22, the deceased fetus with a neurodevelopmental disorder with progressive microcephaly, spasticity, and brain anomalies (NDMSBA). We identified compound heterozygous variants in the *PLAA* gene, resulting in a frameshift and premature termination. Ultrasound indicated intrauterine stillbirth. The mutations were found by trio whole-exome sequencing. Meanwhile, the mutations were absent from the ExAc and HGMD databases. Falik Zaccai et al. [23]. found that plaa-null mice exhibited perinatal lethality. Compound heterozygous variants of LP/P and VUS were detected in the *DYNC2H1*, *PKHD1*, *CAD* and *ECEL1* genes associated with an AR condition in six cases. It is possible that the two variants in the gene act synergistically to result in fetal phenotype. A follow-up parental study to determine the influence of these variants and functional analysis on gene expression will be necessary to assess their association with fetal phenotype.

Of the 11 de novo variants, variants in *FGFR3* genes associated with achondroplasia were identified in three cases. Prenatal ultrasound in all these fetuses revealed severe long bone shortness and constriction of the thorax. This missense variant resulted in a fatal prenatal phenotype of bone dysplasia. The variant identified in the *FGFR3* gene, p.Arg248Cys is identical to the previously reported *FGFR3* gene variant, which has been reported in cases of fetal death [24]. By analyzing the entire exome cohort of fetuses with ultrasound abnormalities, the trio allows for a broader search of disease-causing genes, including de novo variants and recessive genes. Families with de novo variants can be directly informed of the low risk of recurrence in the second pregnancy, despite the possibility of low-level parental mosaicism, which is very rare [25]. These with definitive diagnosed recessive inheritance patterns have high recurrence risks and then had the option for invasive prenatal diagnosis in the subsequent pregnancy to ascertain if the fetus was affected with the same condition.

The relationship between fetal phenotype and genotype was established to clarify that the genetic causes of fetal abnormalities depended on gestational age, the experience of geneticists and the type of imaging utilized. For a fetus with ultrasound abnormalities, it is difficult to identify accurate fetal phenotype due to the difference in gestational age corresponding to the stage of fetal development. Therefore, the diagnosis rate of early pregnancy (GA < 14 W) is low; We can find that the second trimester (GA ≥ 14 W, < 28 W) of pregnancy was the most common among positive diagnosis cases, with a diagnosis rate of 82.1%. In the second trimester, all organs of the fetus have developed, and ultrasound can identify the development of each system, which can better establish the genotype–phenotype association. In particular, the abnormal skeletal system was diagnosed with 35.7% (10/28). Therefore, when systemic abnormalities are found in the second trimester, we cannot ignore the factor of single gene disease. At the same time, there were prominent ultrasound abnormalities at different stages of gestation. Changes in fetal position during the ultrasound examination can make it difficult for the radiologist to identify abnormalities. At the same time, due to differences in the levels of clinical experience, prenatal imaging examinations sometimes cannot accurately identify the clinical phenotype of the fetus. Thus, genetic physicians cannot accurately establish the relationship between genotype and phenotype. It brought the challenge to WES diagnosis and analysis.

There were some limitations to our study. First, as we included only 61 families, the case number in each phenotype category was limited, this cohort of 61 cases could validate the clinical utility of ES but the sample size was insufficient to have a comprehensive evaluation of genetic etiology for deceased fetuses with ultrasound anomalies. secondly, 7 VUS had an effect by interacting with other variants, studies using animal models should be a significant component design to clarify the functional impact of the identified variants, especially the VUS on fetal phenotype.

## Conclusions

In conclusion, ultrasound imaging does not provide an accurate diagnosis due to the lack of adequate phenotypic information in this early stage of fetal development. The application of WES technology has improved the diagnostic rate of fetuses with ultrasound abnormalities and identified the pathogenic effect of monogenic disease. In our cohort, we identified variants in genes known to manifest prenatally and the molecular diagnosis was consistent with the ultrasound findings. By combining this strategy with prenatal imaging, clinicians can help more couples with fetal malformations to identify the underlying genetic etiology and evaluate the recurrence risk. For families with a high risk of recurrence, more accurate counseling and planning can be provided in terms of risk assessment and clinical management.

## Data Availability

Data generated or analyzed during this study are included in this published article. Data supporting the manuscript can be requested from the corresponding author. The web links of the relevant datasets were as follows: hg19 (http://genome.ucsc.edu), Clinvar (http://www.ncbi.nlm.nih.gov/clinvar/), ExAC (http://exac.broad.institute.org/), HGMD (http://www.hgmd.cf.ac.uk/) and OMIM (http://www.omim.org).

## Abbreviations

WES: Whole-exome sequencing
CNV-seq: Copy number variation sequencing
P: Pathogenic
LP: Likely pathogenic
VUS: Variants of uncertain significance
POC: Product of concepts
GATK: Genome Analysis Tool kit
ExAC: Human exons database
HGMD: Human Gene Mutation Database
OMIM: Online Mendelian Inheritance in Man
ACMG: American College of Medical Genetics and Genomics
AD: Autosomal dominant
AR: Autosomal recessive
XLR: X-linked recessive

## References

[1] He M, Du L, Xie H, Zhang L, Gu Y, Lei T, Zheng J, Chen D (2021). The added value of whole-exome sequencing for anomalous fetuses with detailed prenatal ultrasound and postnatal phenotype. Front Genet.

[2] Zhu X, Gao Z, Wang Y, Huang W, Li Q, Jiao Z, Liu N, Kong X (2022). Implementation of trio-based prenatal exome sequencing incorporating splice-site and mitochondrial genome assessment in pregnancies with fetal structural anomalies: prospective cohort study. Ultrasound Obstet Gynecol.

[3] Persson M, Cnattingius S, Villamor E, Soderling J, Pasternak B, Stephansson O, Neovius M (2017). Risk of major congenital malformations in relation to maternal overweight and obesity severity: cohort study of 1.2 million singletons. BMJ.

[4] Zhou Q, Wu SY, Amato K, DiAdamo A, Li P (2016). Spectrum of cytogenomic abnormalities revealed by array comparative genomic hybridization on products of conception culture failure and normal karyotype samples. J Genet Genomics.

[5] van den Veyver IB, Eng CM (2015). Genome-wide sequencing for prenatal detection of fetal single-gene disorders. Cold Spring Harb Perspect Med.

[6] Yates CL, Monaghan KG, Copenheaver D, Retterer K, Scuffins J, Kucera CR, Friedman B, Richard G, Juusola J (2017). Whole-exome sequencing on deceased fetuses with ultrasound anomalies: expanding our knowledge of genetic disease during fetal development. Genet Med.

[7] Richards S, Aziz N, Bale S, Bick D, Das S, Gastier-Foster J, Grody WW, Hegde M, Lyon E, Spector E (2015). Standards and guidelines for the interpretation of sequence variants: a joint consensus recommendation of the American College of Medical Genetics and Genomics and the Association for Molecular Pathology. Genet Med.

[8] Stark Z, Tan TY, Chong B, Brett GR, Yap P, Walsh M, Yeung A, Peters H, Mordaunt D, Cowie S (2016). A prospective evaluation of whole-exome sequencing as a first-tier molecular test in infants with suspected monogenic disorders. Genet Med.

[9] Zhao C, Chai H, Zhou Q, Wen J, Reddy UM, Kastury R, Jiang Y, Mak W, Bale AE, Zhang H (2021). Exome sequencing analysis on products of conception: a cohort study to evaluate clinical utility and genetic etiology for pregnancy loss. Genet Med.

[10] Fu M, Mu S, Wen C, Jiang S, Li L, Meng Y, Peng H (2018). Wholeexome sequencing analysis of products of conception identifies novel mutations associated with missed abortion. Mol Med Rep.

[11] Alamillo CL, Powis Z, Farwell K, Shahmirzadi L, Weltmer EC, Turocy J, Lowe T, Kobelka C, Chen E, Basel D (2015). Exome sequencing positively identified relevant alterations in more than half of cases with an indication of prenatal ultrasound anomalies. Prenat Diagn.

[12] Quinlan-Jones E, Lord J, Williams D, Hamilton S, Marton T, Eberhardt RY, Rinck G, Prigmore E, Keelagher R, McMullan DJ (2019). Molecular autopsy by trio exome sequencing (ES) and postmortem examination in fetuses and neonates with prenatally identified structural anomalies. Genet Med.

[13] Drury S, Williams H, Trump N, Boustred C, Gene GOS, Lench N, Scott RH, Chitty LS (2015). Exome sequencing for prenatal diagnosis of fetuses with sonographic abnormalities. Prenat Diagn.

[14] Mei L, Huang Y, Pan Q, Su W, Quan Y, Liang D, Wu L (2015). Targeted next-generation sequencing identifies novel compound heterozygous mutations of DYNC2H1 in a fetus with short rib-polydactyly syndrome, type III. Clin Chim Acta.

[15] Dagoneau N, Goulet M, Genevieve D, Sznajer Y, Martinovic J, Smithson S, Huber C, Baujat G, Flori E, Tecco L (2009). DYNC2H1 mutations cause asphyxiating thoracic dystrophy and short rib-polydactyly syndrome, type III. Am J Hum Genet.

[16] Schmidts M, Arts HH, Bongers EM, Yap Z, Oud MM, Antony D, Duijkers L, Emes RD, Stalker J, Yntema JB (2013). Exome sequencing identifies DYNC2H1 mutations as a common cause of asphyxiating thoracic dystrophy (Jeune syndrome) without major polydactyly, renal or retinal involvement. J Med Genet.

[17] Rix S, Calmont A, Scambler PJ, Beales PL (2011). An Ift80 mouse model of short rib polydactyly syndromes shows defects in hedgehog signalling without loss or malformation of cilia. Hum Mol Genet.

[18] Ocbina PJ, Eggenschwiler JT, Moskowitz I, Anderson KV (2011). Complex interactions between genes controlling trafficking in primary cilia. Nat Genet.

[19] Grochowski CM, Loomes KM, Spinner NB (2016). Jagged1 (JAG1): Structure, expression, and disease associations. Gene.

[20] Bray SJ (2016). Notch signalling in context. Nat Rev Mol Cell Biol.

[21] Turnpenny PD, Ellard S (2012). Alagille syndrome: pathogenesis, diagnosis and management. Eur J Hum Genet.

[22] Mitchell E, Gilbert M, Loomes KM (2018). Alagille syndrome. Clin Liver Dis.

[23] Falik Zaccai TC, Savitzki D, Zivony-Elboum Y, Vilboux T, Fitts EC, Shoval Y, Kalfon L, Samra N, Keren Z, Gross B (2017). Phospholipase A2-activating protein is associated with a novel form of leukoencephalopathy. Brain.

[24] Lord J, McMullan DJ, Eberhardt RY, Rinck G, Hamilton SJ, Quinlan-Jones E, Prigmore E, Keelagher R, Best SK, Carey GK (2019). Prenatal exome sequencing analysis in fetal structural anomalies detected by ultrasonography (PAGE): a cohort study. Lancet.

[25] Gambin T, Liu Q, Karolak JA, Grochowski CM, Xie NG, Wu LR, Yan YH, Cao Y, Coban Akdemir ZH, Wilson TA (2020). Low-level parental somatic mosaic SNVs in exomes from a large cohort of trios with diverse suspected Mendelian conditions. Genet Med.

